# Adverse Maternal and Neonatal Outcomes in Women With Elevated Intrapartum Temperature Complicated by Histological Chorioamnionitis at Term: A Propensity-Score Matched Study

**DOI:** 10.3389/fped.2021.654596

**Published:** 2021-04-22

**Authors:** Yingzhou Ge, Chen Zhang, Yanqing Cai, Hefeng Huang

**Affiliations:** ^1^International Peace Maternity and Child Health Hospital, School of Medicine, Shanghai Jiao Tong University, Shanghai, China; ^2^Shanghai Key Laboratory of Embryo Original Diseases, Shanghai, China; ^3^Research Units of Embryo Original Diseases, Chinese Academy of Medical Sciences, Shanghai, China; ^4^Obstetrics and Gynecology Hospital of Fudan University, Shanghai, China

**Keywords:** histological chorioamnionitis, intrapartum hyperthermia, NICU admission, neonatal sepsis, prolonged hospitalization, puerperal morbidity

## Abstract

**Background:** Elevated intrapartum temperature has been widely proven to be associated with adverse clinical outcomes in both mothers and neonates. Histological chorioamnionitis (HCA), the inflammation of chorion and amniotic membranes, is commonly observed in those with elevated intrapartum temperature. Thus, we aimed to explore whether the combination of HCA would further affect the pregnancy outcomes in those with intrapartum temperature ≥ 37.5°C.

**Methods:** This retrospective cohort study was conducted at the International Peace Maternity and Child Health Hospital (IPMCH), including all full-term women with intrapartum temperature ≥ 37.5°C from Jan 2017 to Jan 2019. Patients were divided in to HCA group or control group according to placental pathology results, and we used 1:1 propensity score matching (PSM) to reduce the effects of potential confounding factors between the two groups. Univariate and multivariable logistic regression were used to identify the association between HCA and different adverse maternal and neonatal outcomes.

**Results:** We formed a propensity-score matched cohort containing 464 women in each group. Higher positive rate of mycoplasma (14.01% vs. 7.33%, *p* = 0.001) was found in the vaginal secretion culture of women in the HCA group. After adjusting for various baseline clinical characteristics, women with HCA were more likely to end their delivery by cesarean section (AOR = 1.55, 95% CI: 1.05–2.28), and puerperal morbidity (AOR = 2.77, 95% CI: 1.44–5.33) as well as prolonged hospitalization (AOR = 1.56, 95% CI: 1.12–2.17) were more likely to be observed in the HCA group. The existence of HCA might also be associated with neonatal sepsis (AOR = 2.83, 95% CI: 1.14–7.04) and NICU admission (AOR = 1.40, 95% CI: 1.04–1.87) in newborns. In the study on the impact of different stages of HCA, we found that both maternal and neonatal outcomes would not be affected by mild HCA (stage I), while HCA of stage III was associated with increased need for neonatal respiratory support and elevated likelihood of prolonged hospitalization in neonates.

**Conclusions:** Elevated intrapartum temperature complicated by HCA might be related to the elevated occurrence of several adverse maternal and neonatal outcomes, except those with HCA of stage I. Advanced HCA stage correlated with a worse prognosis.

## Introduction

Elevation of body temperature is a common phenomenon during labor, which can be caused by both infectious and noninfectious factors (such as dehydration, epidural anesthesia, the use of prostaglandins for labor induction, and increased ambient temperature) (1, 2). Most studies currently use temperature ≥ 38°C as the standard for intrapartum hyperthermia (3), but the condition of 37.5°C ≤ temperature <38°C during labor is also considered as borderline fever or sub febrile status by some studies (4). Therefore, the normal temperature should be <37.5°C, and once the intrapartum temperature continues to be ≥37.5°C, it can be regarded as elevated intrapartum temperature. Currently, it has been widely confirmed that elevated intrapartum temperature is associated with adverse maternal and neonatal outcomes, including primary cesarean section, neonatal sepsis, neonatal encephalopathy, etc (5, 6).

Chorioamnionitis refers to acute inflammation of the membranes and chorion of the placenta, and is strongly associated with microbial invasion (7, 8). According to different diagnostic criteria, chorioamnionitis can be divided into clinical chorioamnionitis (CCA) and histological chorioamnionitis (HCA). The diagnosis of CCA is usually based on Gibbs criteria and its variations, that is, intrapartum temperature ≥37.8°C along with at least two additional signs (different variations have different thresholds for body temperature and number of clinical manifestations): uterine tenderness, maternal tachycardia, fetal tachycardia, foul/purulent vaginal discharge and maternal leukocytosis (WBC count ≥15.00 × 10^9^/L) (9, 10). HCA, also called as subclinical chorioamnionitis, usually has no obvious symptoms, with elevated body temperature as its only clinical manifestation in most cases. The diagnosis of HCA is based entirely on pathological examination of the placenta (11, 12). It occurs in 40–70% of preterm births and 1–13% of term births (8, 13), and could lead to various adverse maternal outcomes and a poorer prognosis in neonates (14–17).

Due to the strong association between HCA and preterm birth, most of the current studies on the effect of HCA on pregnancy outcomes were carried out in a preterm population, while there are limited studies focusing on pregnancy outcomes at term. At the same time, although the separate effects of elevated intrapartum temperature and HCA on maternal and neonatal outcomes have been widely confirmed, few studies have compared the differences between elevated intrapartum temperature alone and those complicated by HCA. Therefore, we designed this retrospective cohort study to explore whether the combination of HCA will further worsen the prognosis of mothers and newborns in full-term women with elevated intrapartum temperature, in order to provide scientific support for their clinical management and treatment.

## Materials and Methods

### Study Population and Methods

This single-center, retrospective, 1:1 matched cohort study was conducted from Jan 2017 to Jan 2019, at the International Peace Maternity and Child Health Hospital (IPMCH), which is a tertiary specialized hospital of obstetrics and gynecology and accounts for 10–20% of births in Shanghai, China. We included all women with a record of elevated temperature (at least one temperature record of ≥37.5°C) after entering active labor (cervical dilatation ≥4 cm). Exclusion criteria included preterm birth (<37 weeks), multiple pregnancy, confirmed infection outside the uterine cavity, trial of labor after cesarean section (TOLAC), fetal structural or chromosomal abnormalities, and missing or untraceable clinical data.

All the cohort data are available at the Res Man Manager of Chinese Clinical Trial Registry (www.medresman.org), and the registration number is ChiCTR2000038345. Ethical approval was given by the Institutional Review Board of the IMPCH (No. GKLW 2013-51).

### Examination

For women with intrapartum temperature ≥37.5°C, placental pathological examinations are routinely performed in our hospital to provide guidance for the subsequent treatment. This process is completed by the Pathology Department of the IPMCH. Based on the results of placental pathology, we further divided those who met the inclusion and exclusion criteria into an HCA group and a control group. The diagnosis of HCA and the assessment of its severity were made according to the Blanc classification: polymorphonuclear lymphocytes infiltrate the subchorion area (stage I), infiltration of the chorionic membrane (stage II), and infiltration of both the chorionic and amniotic membranes (stage III) (18).

For all women, complete blood count and C-reactive protein (CRP) levels were measured after the detection of an elevated temperature. Sampling of vaginal secretions should also be completed at that time, and samples should be sent for routine culture and anaerobic culture as soon as possible. For their newborns, complete blood count, CRP levels and procalcitonin (PCT) levels were routinely tested on the first day after delivery, and for those admitted to NICU, blood culture is routinely performed.

### Outcomes

All data were collected from the electronic medical record System. The information collected for each woman included socio-demographic characteristics, reproductive history, antepartum and intrapartum factors, the results of laboratory tests performed at the time of elevated temperature, and pregnancy outcomes. Data collected for each newborn included birth records, the results of laboratory tests performed on the first day after delivery and neonatal complications.

Adverse maternal outcomes were as follows: assisted vaginal delivery (forceps or vacuum), cesarean section, placental adhesion, cervix or vagina laceration, postpartum hemorrhage, puerperal morbidity, late postpartum hemorrhage, admittance to intensive care unit (ICU), and hospitalization time (from entering active labor to discharge) ≥7 days. Puerperal morbidity means after 24 h and within 10 days after childbirth, the oral temperature reached 38°C for at least twice with an intervals of 4 h, which is mostly caused by puerperal infection.

Adverse neonatal outcomes included 5-min Apgar score <7, small for gestational age (SGA), birth trauma (head trauma, skin damage, etc.), neonatal jaundice (19), neurological morbidity (neonatal epilepsy, cerebral palsy, etc.), respiratory distress syndrome (RDS), pneumonia, the need for respiratory assistance/support, necrotising enterocolitis (NEC), neonatal sepsis, blood transfusion, admittance to neonatal intensive care unit (NICU), hospitalization time ≥7 days, and neonatal death. Neonatal sepsis was diagnosed when pathogenic bacteria were observed through blood culture or sterile body cavity culture or conditional pathogenic bacteria was observed through blood culture, and was re-observed in another blood culture, sterile body cavity culture or catheter head culture.

### Statistical Analysis

To reduce the effects of potential confounding factors between the two groups, we first screened out those who met the diagnosis of CCA according to the Gibbs criteria. Secondly, a 1:1 propensity score matching (PSM) was used to identify the rest non-CCA women with similar baseline clinical characteristics, including maternal age, pre-pregnancy BMI, educational levels, parity, gestational age, mode of conception, gestational diabetes mellitus (GDM), hypertensive disorders of pregnancy, hypothyroidism, premature rupture of membranes (PROM), duration from rupture of membranes (ROM) to delivery, presence of meconium-stained amniotic fluid, maximal intrapartum temperature, duration of fever, intrapartum antibiotic use, and fetal sex. All the above variables were entered into a multivariate logistic regression model to estimate propensity scores, and matching was performed by using a 1:1 nearest neighbor matching algorithm with a caliper width equal to 0.1 without replacement. Standardized mean differences (SMDs) and *p*-values before and after PSM of each variable were calculated, and a SMD of <10% along with a *p*-value more than 0.05 indicated a relatively small imbalance.

The Kolmogorov–Smirnov test was used to assess the normal distribution. Normally distributed variables are expressed as mean ± standard deviation (SD), and non-normally distributed variables are expressed as median (interquartile range, IQR). Continuous data were compared by using either Student's *t*-tests or Mann-Whitney U tests, and categorical data were compared by using either chi-Squared tests or Fisher's exact test. To identify the association between HCA and different adverse outcomes, univariate and multivariable logistic regression analyses were used, and odds ratios (ORs) were adjusted for all variables involved in PSM. Sensitivity analysis and subgroup analysis were used to assess the difference in certain maternal and neonatal outcomes in each subgroup between the matched two groups to identify potential bias. P for interaction <0.1 was considered to indicate significant interactions in the subgroups. A two-sided *p*-value of <0.05 was considered statistically significant. All statistical analyses were performed by using SPSS (version 22.0; SPSS, Inc., Chicago, IL, USA) and R statistical software version 4.0.2 (packages “MatchIt,” “tableone,” “ggplot2,” “survey,” “forestplot”).

## Results

### Baseline Clinical Characteristics

**Figure 1:**
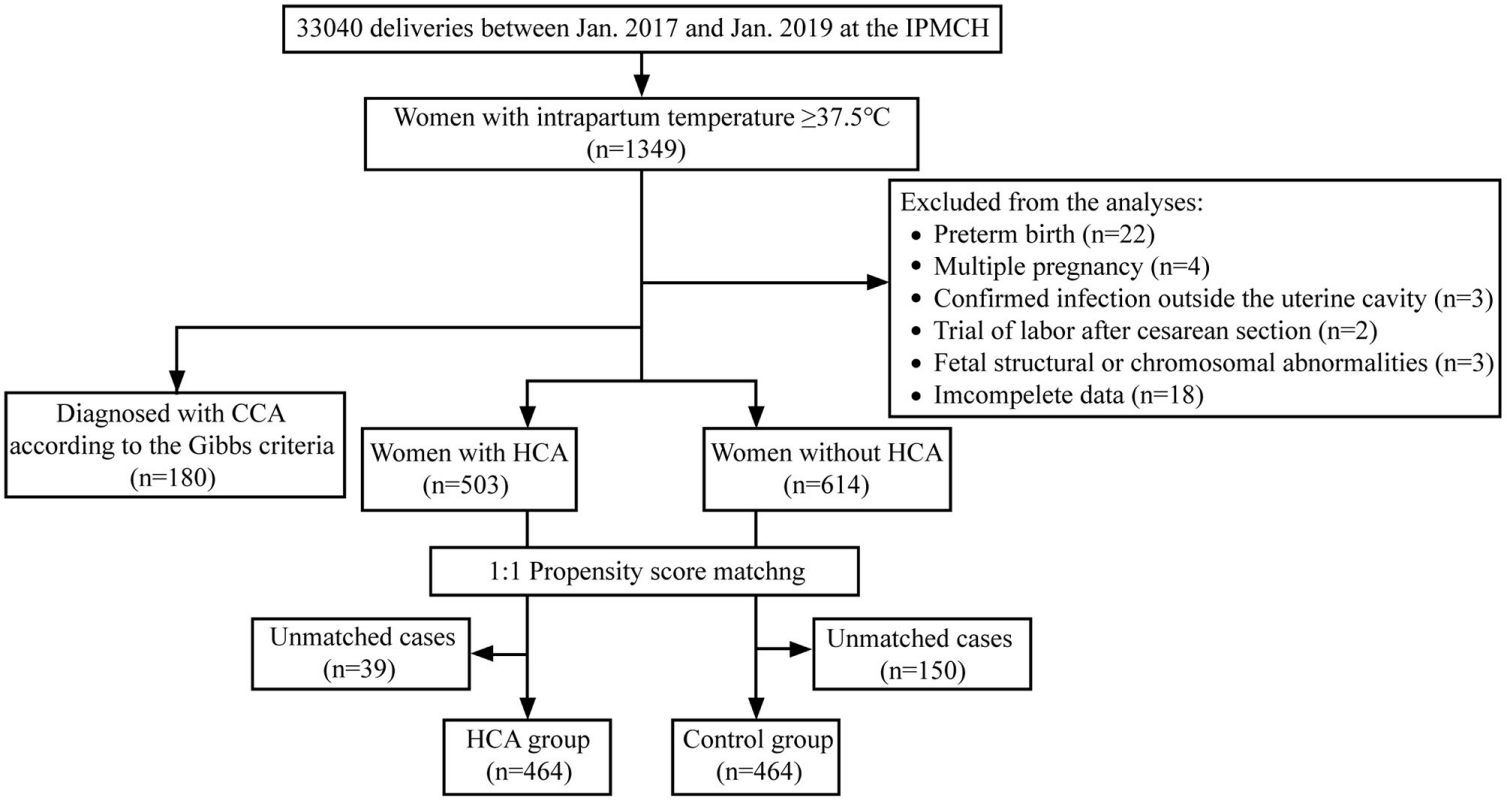
Flow chart.

A total of 33,040 women gave birth in our hospital during the study period, and 1,349 women were detected with elevated body temperature after entering active labor. Among them, 52 were excluded according to the exclusion criteria, and the rest were further classified as with HCA (*n* = 503) or without HCA (*n* = 614). Finally, we constructed a propensity-score-matched population of 464 women in each group (flow chart shown in Figure 1).

The comparisons of the clinical features between the two groups before and after PSM were shown in Table 1. Before PSM, the proportion of women with GDM (13.1 vs. 6.5%, *p* < 0.001), PROM (65.8 vs. 53.9%, *p* < 0.001), and intrapartum temperature <38°C (34.0 vs. 25.9%, *p* = 0.003) in the HCA group was higher than that in the control group. After matching, the *p*-values of all variables were > 0.05, and the SMD of all variables were <10%, indicating no significant differences between the two groups.

**Table 1:** Baseline clinical characteristics before and after propensity-score matching^a^.

**Variables**	**Before matching**				**After matching**			
	**Control group *n* = 614**	**HCA group *n* = 503**	**SMD**	***P*-value**	**Control group *n* = 464**	**HCA group *n* = 464**	**SMD**	***P*-value**
**GENERAL INFORMATION**								
**Maternal age (year)**								
**Mean** **±** **SD**	30.09 ± 0.27	30.23 ± 0.30	0.040	0.506	30.19 ± 0.30	30.16 ± 0.31	0.002	0.977
**Distribution**			0.021	0.724			0.014	0.828
<35	549 (89.4)	453 (90.1)			416 (89.7)	418 (90.1)		
≥35	65 (10.6)	50 (9.9)			48 (10.3)	46 (9.9)		
**Pre-pregancancy BMI (kg/m**^**2**^**)**								
**Mean** **±** **SD**	20.99 ± 0.20	21.14 ± 0.24	0.059	0.327	21.05 ± 0.23	21.09 ± 0.26	0.011	0.870
**Distribution**			0.080	0.412			0.025	0.932
<18.5	96 (15.6)	72 (14.3)			72 (15.5)	69 (14.9)		
18.5–23.9	448 (73.0)	361 (71.8)			335 (72.2)	335 (72.2)		
≥24	70 (11.4)	70 (13.9)			57 (12.3)	60 (12.9)		
**Education level**			0.068	0.535			0.050	0.746
Bachelor or above	543 (88.4)	455 (90.5)			416 (89.7)	419 (90.3)		
High school	59 (9.6)	39 (7.8)			41 (8.8)	36 (7.8)		
Middle school or below	12 (2.0)	9 (1.8)			7 (1.5)	9 (1.9)		
**OBSTETRIC CHARACTERISTICS**								
**Parity**			0.062	0.303			0.015	0.815
Primiparous	553 (90.1)	462 (91.9)			425 (91.6)	423 (91.2)		
Multiparous	61 (9.9)	41 (8.2)			39 (8.4)	41 (8.8)		
**Gestational age (weeks)**	39.41 ± 0.09	39.51 ± 0.12	0.081	0.175	39.47 ± 0.10	39.48 ± 0.13	0.006	0.924
**ART**			0.025	0.677			0.017	0.798
No	566 (92.2)	467 (92.8)			432 (93.1)	430 (92.7)		
Yes	48 (7.8)	36 (7.2)			32 (6.9)	34 (7.3)		
**GDM**			0.223	<0.001			0.024	0.715
No	574 (93.5)	437 (86.9)			429 (92.5)	426 (91.8)		
Yes	40 (6.5)	66 (13.1)			35 (7.5)	38 (8.2)		
**Hypertensive disorders of pregnancy**			0.010	0.864			0.012	0.855
No	592 (96.4)	484 (96.2)			449 (96.8)	448 (96.6)		
Yes	22 (3.6)	19 (3.1)			15 (3.2)	16 (3.45)		
**Hypothyroidism**			0.064	0.288			0.028	0.671
No	585 (95.3)	472 (93.8)			439 (94.6)	436 (94.0)		
Yes	29 (4.7)	31 (6.2)			25 (5.4)	28 (6.0)		
**PROM**			0.244	<0.001			0.004	0.946
No	283 (46.1)	172 (34.2)			168 (36.2)	169 (36.4)		
Yes	331 (53.9)	331 (65.8)			296 (63.8)	295 (63.6)		
**Duration of ROM (hours)**			0.130	0.097			0.060	0.655
No	111 (18.1)	82 (16.3)			82 (17.7)	78 (16.8)		
<18	372 (60.6)	286 (56.9)			273 (58.8)	265 (57.1)		
≥18	131 (21.3)	135 (26.8)			109 (23.5)	121 (26.1)		
**Meconium-stained amniotic fluid**			0.046	0.447			0.019	0.772
No	446 (72.6)	355 (70.6)			332 (71.6)	328 (70.7)		
Yes	168 (27.4)	148 (29.4)			132 (28.5)	136 (29.3)		
**Intrapartum temperature (°C)**			0.178	0.003			0.014	0.831
37.5–37.9	455 (74.1)	332 (66.0)			324 (69.8)	321 (69.2)		
≥38	159 (25.9)	171 (34.0)			140 (30.2)	143 (30.8)		
**Duration of fever (hours)**			0.036	0.836			0.022	0.946
<2	400 (65.2)	336 (66.8)			307 (66.2)	305 (65.7)		
2–4	151 (24.6)	119 (23.7)			114 (24.6)	113 (24.4)		
≥4	63 (10.3)	48 (9.5)			43 (9.3)	46 (9.9)		
**Intrapartum antibiotic use**			0.066	0.270			0.047	0.470
No	306 (49.8)	234 (46.5)			231 (49.8)	220 (47.4)		
Yes	308 (50.2)	269 (53.5)			233 (50.2)	244 (52.6)		
**Fetal sex**			0.005	0.939			0.004	0.948
Boy	331 (53.9)	270 (53.7)			246 (53.0)	245 (52.8)		
Girl	283 (46.1)	233 (46.3)			218 (47.0)	219 (47.2)		

*HCA, histological chorioamnionitis; SMD, standardized mean difference; SD, standard deviation; BMI, body mass index; ART, assisted reproductive technology; GDM, gestational diabetes mellitus; PROM, premature rupture of membranes; ROM, rupture of membranes*.

a *Data is presented as mean ± SD or frequency (percentage)*.

### Maternal Clinical Outcomes and Laboratory Results

**Figure 2:**
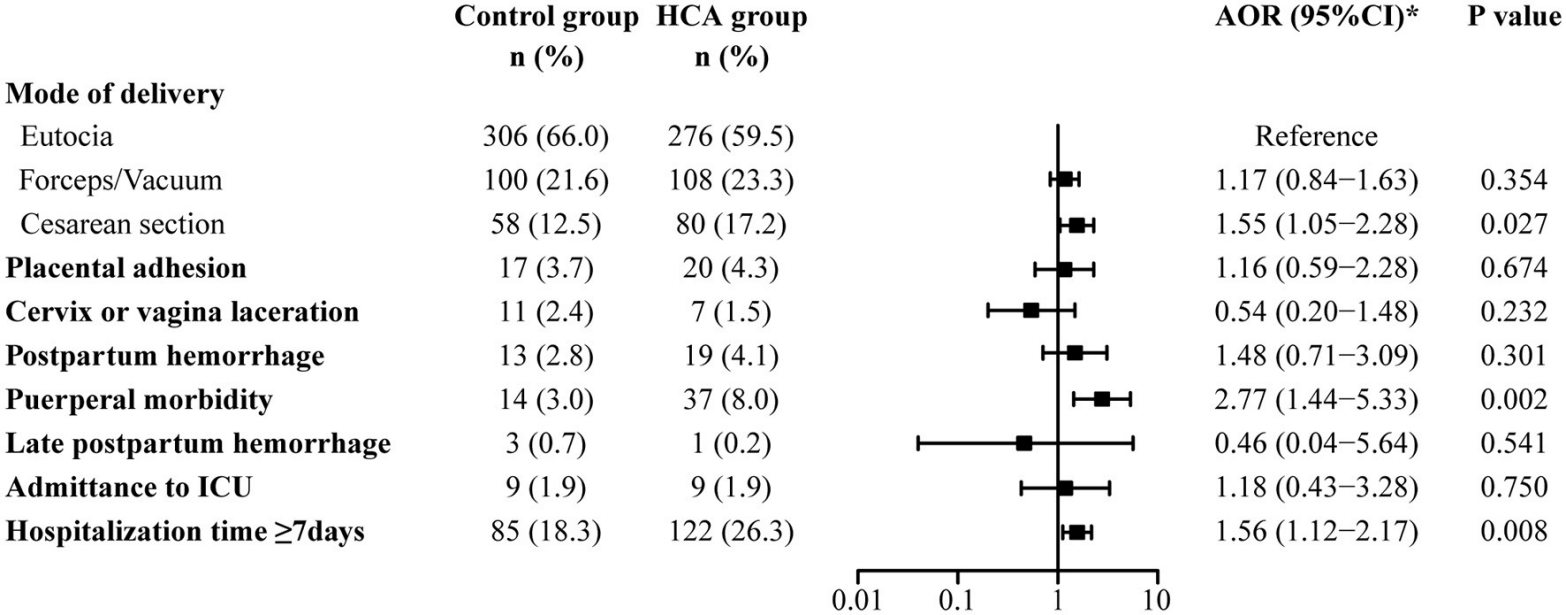
Maternal outcomes in the propensity-score matched cohort. ^*^Adjusted for all baseline clinical factors mentioned in PSM.

Univariate and multivariable logistic regression analyses were used to assess adverse maternal outcomes (Supplementary Table 1, Figure 2). Compared with the control group, women in the HCA group were more likely to end their delivery by cesarean section (17.2 vs. 12.5%, AOR = 1.55, 95% CI: 1.05–2.28). Puerperal morbidity (8.0 vs. 3.0%, AOR = 2.77, 95% CI: 1.44–5.33) and prolonged hospitalization (≥7 days) (26.3 vs. 18.3%, AOR = 1.56, 95% CI: 1.12–2.17) were more likely to be observed in women with HCA. There was no significant difference in the rate of assisted vaginal delivery, placental adhesion, cervix or vagina laceration, postpartum hemorrhage or admittance to ICU between the two groups.

### Neonatal Clinical Outcomes and Laboratory Results

**Figure 3:**
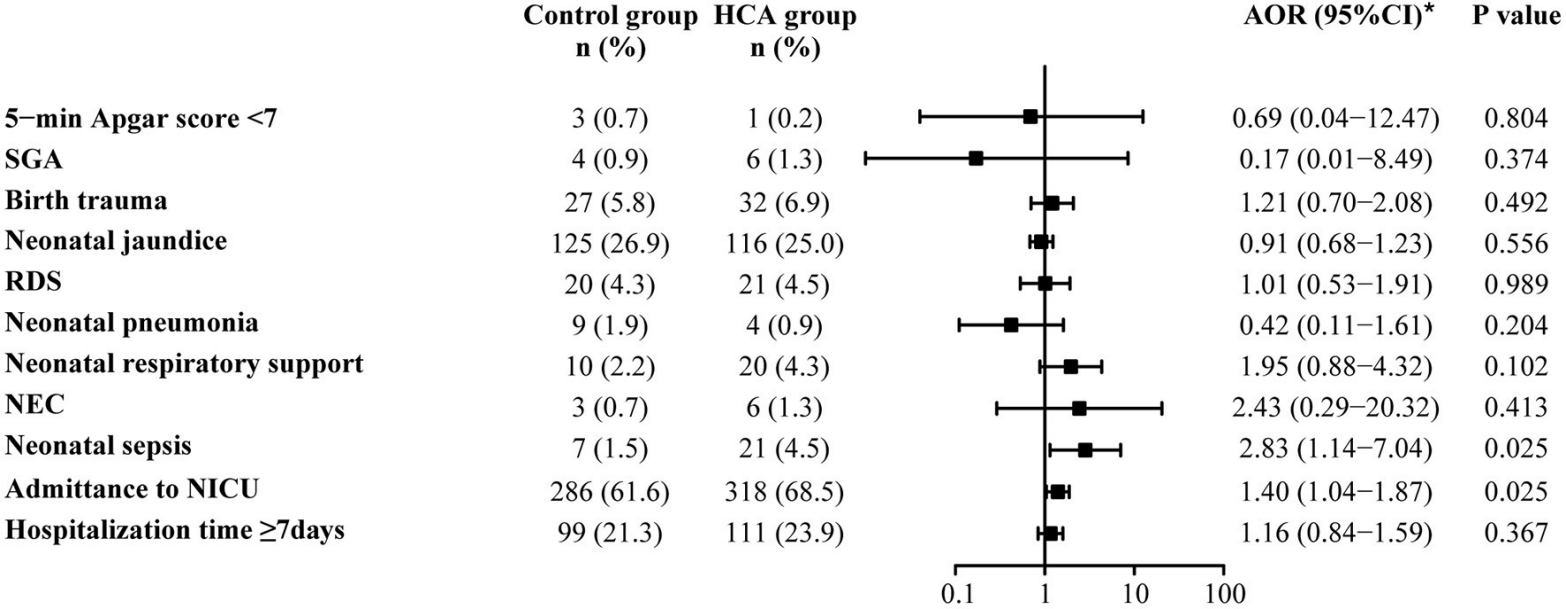
Neonatal outcomes in the propensity-score matched cohort. *Adjusted for all baseline clinical factors mentioned in PSM. There was 1 neonate in the control group and 3 in the HCA group who underwent blood transfusion, but the adjusted OR is not applicable, thus the data not shown in the figure.

Adverse neonatal outcomes were also assessed by using univariate and multivariable logistic regression analyses. After adjusting for all baseline clinical characteristics mentioned in PSM, we found that HCA seemed to be associated with admittance to NICU (68.5 vs. 61.6%, AOR = 1.40, 95% CI: 1.04–1.87) and neonatal sepsis (4.5 vs. 1.5%, AOR = 2.83, 95% CI: 1.14–7.04). No differences were found between the two groups in the remaining adverse outcomes (Supplementary Table 2, Figure 3).

Neonates of women with HCA had higher level of WBC count (28.10 vs. 26.80 × 10^9^/L, *p* = 0.019). Neutrophil count, CRP level and PCT level showed no significant differences between the two groups (Table 2).

**Table 2:** Maternal and neonatal laboratory examination results in the propensity-score matched cohort^a^.

**Variables**	**Control group *n* = 464**	**HCA group *n* = 464**	***P*-value**
**Maternal blood test**			
Maternal WBC count (×10^9^/L)	14.60 (12.23–17.30)	15.20 (12.70–17.80)	0.063
Maternal WBC count ≥ 15.00 × 10^9^/L	218 (47.0)	245 (52.8)	0.076
Maternal netrophil count (×10^9^/L)	12.75 (10.38–15.17)	12.98 (10.51–15.79)	0.096
Maternal CRP (mg/L)	10.00 (6.00–18.75)	15.00 (8.00–28.00)	<0.001
Maternal CRP ≥ 10mg/L	243 (52.4)	329 (70.9)	<0.001
**Maternal vaginal secretion cultivation**			
Mycoplasma (+)	34 (7.3)	65 (14.0)	0.001
GBS (+)	31 (6.7)	33 (7.1)	0.796
Gram-positive bacteria (+)	15 (3.2)	26 (5.6)	0.079
Gram-negative bacteria (+)	91 (19.6)	74 (16.0)	0.144
**Neonatal blood test**			
Neonatal WBC count (×10^9^/L)	26.80 (22.60–31.78)	28.10 (23.73–33.60)	0.019
Maternal WBC count ≥ 25.00 × 10^9^/L	289 (62.3)	316 (68.1)	0.063
Neonatal netrophil count (×10^9^/L)	19.86 (16.31–24.30)	20.65 (16.66–25.35)	0.113
Neonatal CRP (mg/L)	5.00 (3.00–10.00)	6.00 (4.00–11.00)	0.067
Neonatal CRP ≥ 8mg/L	162 (34.9)	188 (40.5)	0.078
Neonatal PCT (ng/mL)	0.12 (0.09–0.18)	0.13 (0.10–0.19)	0.098

*HCA, histological chorioamnionitis; WBC, white blood cells; CRP, C- reactive protein; GBS, group B streptococcus; PCT, procalcitonin*.

a *Data is presented as median (interquartile range) or frequency (percentage)*.

### Subgroup Analysis

**Figure 4:**
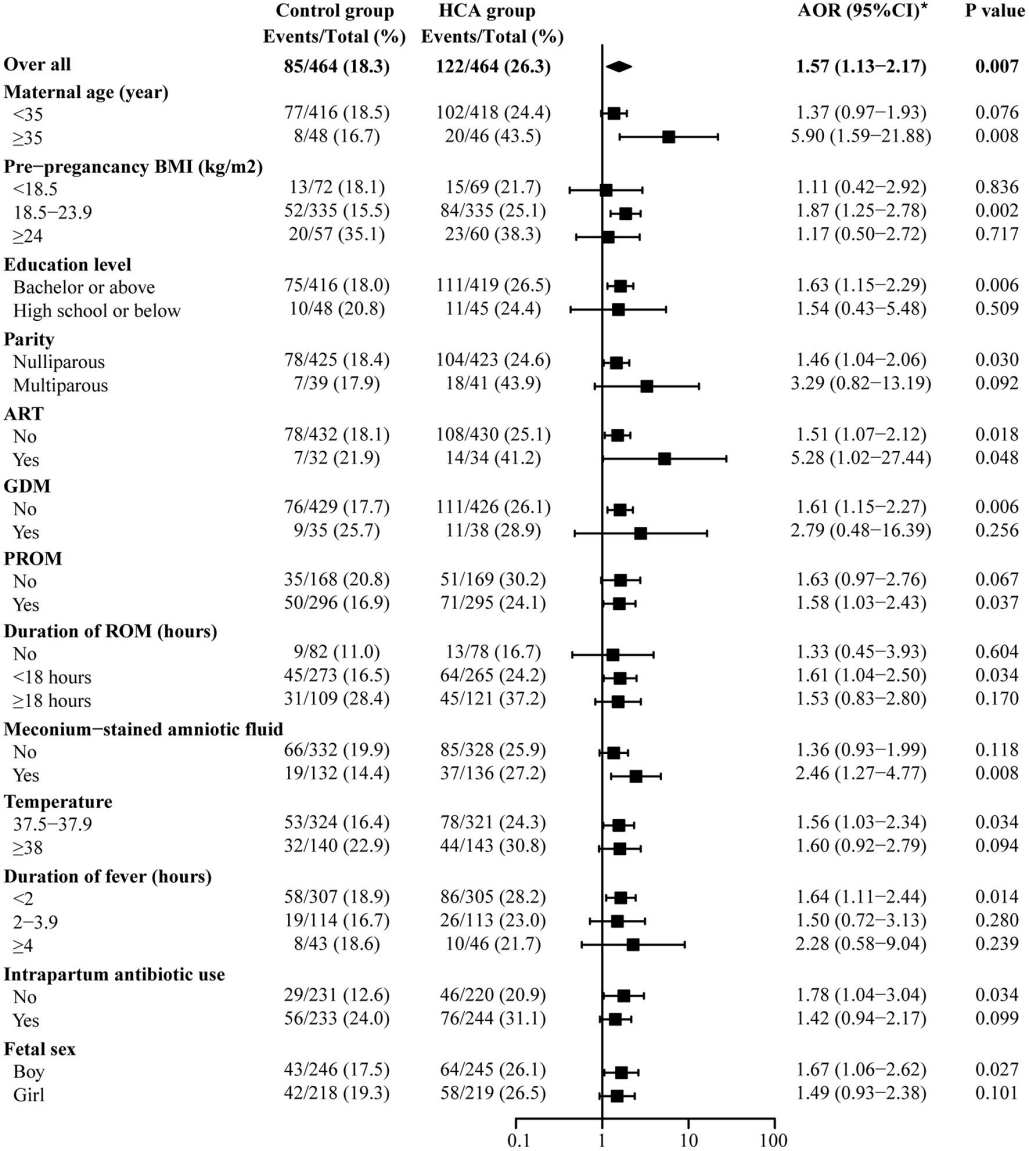
Subgroup analysis of maternal prolonged hospitalization. *Adjusted for all baseline clinical factors mentioned in PSM. The adjusted OR of hypertensive disorders of pregnancy and hypothyroidism are not applicable, thus the data not shown in the figure.

Figure 4 shows the results of the maternal prolonged hospitalization subgroup analysis. For those with HCA, an increased proportion of long-term hospitalization was found in all subgroups. Women in the HCA cohort with advanced age were more likely to experience prolonged hospitalization [AOR 5.90 (1.59–21.88) vs. 1.37 (0.97–1.93), *p* for interaction = 0.054] in comparison to those under 35 years old.

**Figure 5:**
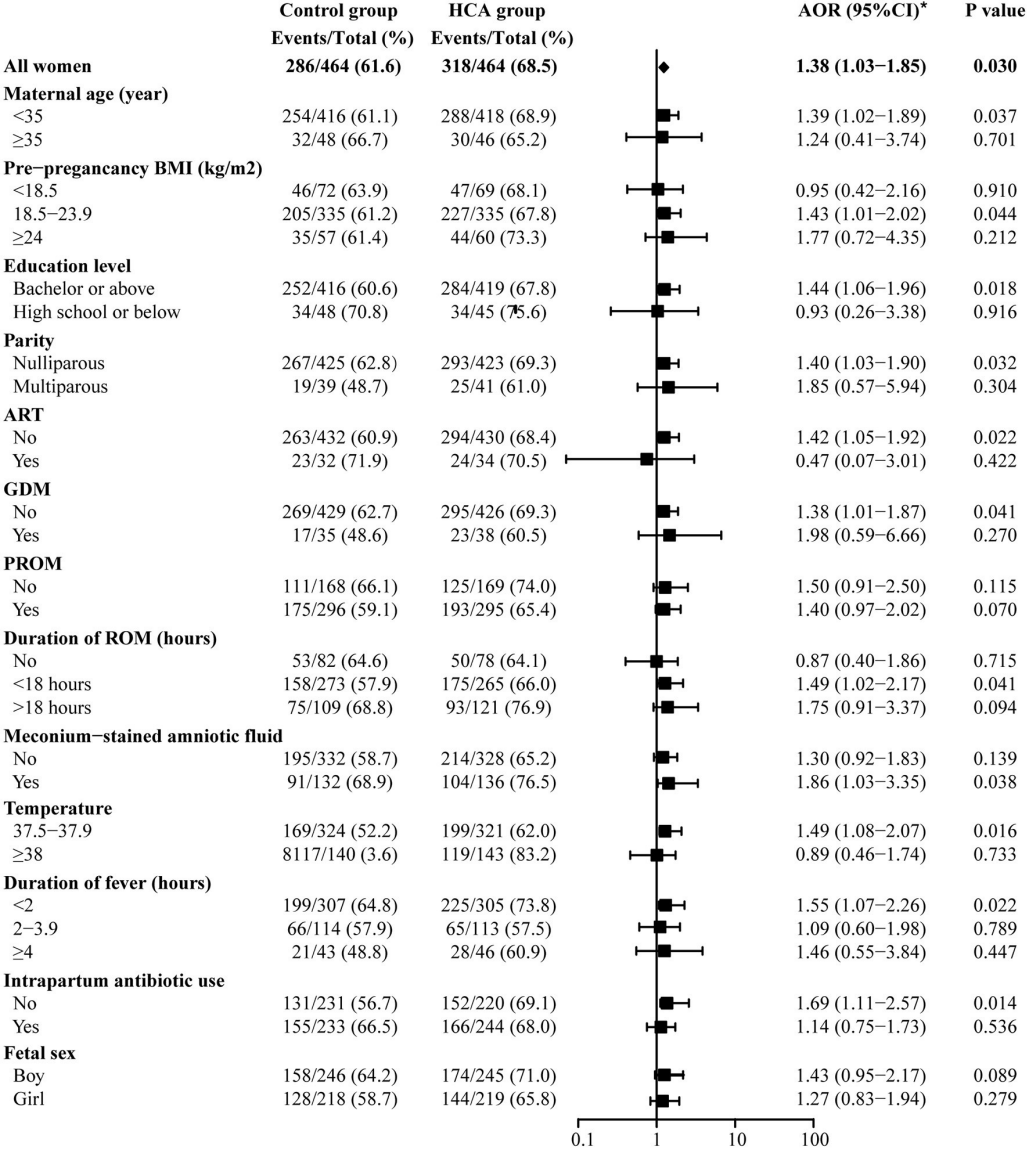
Subgroup analysis off admittance to NICU. *Adjusted for all baseline clinical factors mentioned in PSM. The adjusted OR of hypertensive disorders of pregnancy and hypothyroidism are not applicable, thus the data not shown in the figure.

Figure 5 shows the results of the NICU-admittance subgroup analysis. The results suggest that admittance to NICU was more likely to be observed in the HCA group among most of the subgroups, except those with pre-pregnancy BMI <18.5 (AOR = 0.95, 95% CI: 0.42–2.16), low educational level (AOR = 0.93, 95% CI: 0.26–3.38), intrapartum temperature ≥38°C (AOR = 0.89, 95% CI: 0.46–1.74), conceived through ART (AOR = 0.47, 95% CI: 0.07–3.01) or those without ROM (AOR = 0.87, 95% CI: 0.40–1.86). Meanwhile, for neonates in the HCA group, the maternal use of antibiotics during labor might decrease its likelihood of NICU admission [AOR 1.14 (0.75–1.73) vs. 1.69 (1.11–2.57), *p* for interaction = 0.096].

Since the standard for intrapartum hyperthermia is temperature ≥38°C, we further analyzed that in the subgroup of intrapartum temperature ≥38°C, whether HCA would lead to adverse pregnancy outcomes. The results are shown in Supplementary Table 3 and Supplementary Table 4. We found that in such subgroup, the existence of HCA was associated with puerperal morbidity (AOR = 5.37, 95% CI: 1.58–18.27, *p* = 0.007) in mothers and higher propotion of neonatal sepsis (OR = 9.34, 95% CI: 1.17–74.70, *p* = 0.035) in newborns.

Meanwhile, there were another 180 women who met the diagnostic criteria of CCA in our study population, thus, we compared the differences in pregnancy outcomes between the CCA group and the control group (without PSM). The results are shown in Supplementary Table 5 and Supplementary Table 6. Similar to HCA, CCA was also closely related to adverse pregnancy outcomes. Women in the CCA group were more likely to end their delivery by cesarean section (AOR = 1.94, 95% CI: 1.08–3.49, *p* = 0.027) and cervix or vagina laceration (AOR = 3.18, 95% CI: 1.02–9.94, *p* = 0.046). And the probability of neonatal sepsis was elevated in the CCA group (AOR = 3.95, 95% CI: 1.01–15.55, *p* = 0.049).

### Maternal and Neonatal Outcomes of Different Stages of HCA

According to the results of placental pathology, 139, 209 and 116 women were evaluated as HCA stage I, stage II and stage III, respectively. After adjusting for all baseline clinical characteristics (those used for PSM), we did not find an association between adverse maternal outcomes and HCA stage I. Women of HCA stage II were more likely to develop puerperal morbidity (AOR = 4.46, 95% CI: 2.05–9.74). Those with HCA stage III showed higher likelihood of cesarean section (AOR = 2.22, 95% CI: 1.24–3.97) and prolonged hospitalization (AOR = 3.34, 95% CI: 2.08–5.37) (Table 3).

**Table 3:** Multivariable regression analysis of maternal outcomes after adjustment.

	**Control** **group** ***n* = 464**	**HCA** **stage I** ***n* = 139**	**HCA** **stage II** ***n* = 209**	**HCA** **stage III *n* = 116**	**Control vs. HCA stage I**		**Control vs. HCA stage II**		**Control vs. HCA stage III**	
					**AOR (95% CI)^a^**	***P*-value**	**AOR (95% CI)^*a*^**	***P*-value**	**AOR (95% CI)^a^**	***P*-value**
Mode of delivery										
Eutocia	306 (66.0)	89 (64.0)	124 (59.3)	63 (54.3)	Reference		Reference		Reference	
Forceps/Vacuum	100 (21.6)	28 (20.1)	51 (24.4)	29 (25.0)	0.97 (0.58–1.63)	0.920	1.22 (0.81–1.86)	0.346	1.37 (0.80–2.33)	0.249
Cesarean section	58 (12.5)	22 (15.8)	34 (16.3)	24 (20.7)	1.35 (0.77–2.39)	0.300	1.40 (0.86–2.30)	0.176	2.22 (1.24–3.97)	0.007
Placental adhesion	17 (3.7)	2 (1.4)	13 (6.2)	5 (4.3)	0.40 (0.09–1.87)	0.247	1.59 (0.74–3.45)	0.239	1.09 (0.37–3.18)	0.882
Cervix or vagina laceration	11 (2.4)	2 (1.4)	2 (1.0)	3 (2.6)	0.53 (0.11–2.53)	0.424	0.33 (0.07–1.63)	0.175	0.84 (0.19–3.68)	0.817
PPH	13 (2.8)	5 (3.6)	10 (4.8)	4 (3.5)	1.30 (0.42–4.01)	0.653	1.75 (0.72–4.26)	0.219	1.16 (0.33–4.10)	0.815
Puerperal morbidity	14 (3.0)	7 (5.0)	22 (10.5)	8 (6.9)	1.85 (0.70–4.92)	0.218	4.46 (2.05–9.74)	<0.001	2.30 (0.89–5.91)	0.085
Late PPH	3 (0.7)	0 (0)	0 (0)	1 (0.9)	NA	NA	NA	NA	1.69 (0.11–25.98)	0.706
Admittance to ICU	9 (1.9)	4 (2.9)	3 (1.4)	2 (1.72)	1.81 (0.41–7.97)	0.432	1.23 (0.26–5.72)	0.793	1.54 (0.24–9.85)	0.650
Hospitalization time≥7days	85 (18.3)	24 (17.3)	50 (23.9)	48 (41.4)	0.94 (0.55–1.61)	0.831	1.28 (0.84–1.96)	0.254	3.34 (2.08–5.37)	<0.001

*HCA, histological chorioamnionitis; AOR, adjusted odds ratio; CI, confidence interval; ICU, intensive care unit; NA, not applicable*.

a *Adjusted for all baseline clinical factors mentioned in PSM*.

Compared with those in the control group, neonates of women with HCA stage I were less likely to develop neonatal jaundice (AOR = 0.47, 95% CI: 0.28–0.80) and showed a lower likelihood of prolonged hospitalization (AOR = 0.47, 95% CI: 0.26–0.85). As for neonates in the HCA stage II group, higher likelihood of neonatal sepsis (AOR = 3.43, 95% CI: 1.21–9.69) and admittance to NICU (AOR = 1.55, 95% CI: 1.05–2.27) were observed. And those in the HCA stage III group showed increased need for respiratory support (AOR = 4.08, 95% CI: 1.44–11.57), and elevated proportion of neonatal sepsis (AOR = 4.57, 95% CI: 1.34–15.61), admittance to NICU (AOR = 3.35, 95% CI: 1.92–5.84) and prolonged hospitalization (AOR = 2.05, 95% CI: 1.29–3.27) (Table 4).

**Table 4:** Multivariable regression analysis of neonatal outcomes after adjustment.

	**Control** **group** ***n* = 464**	**HCA** **stage I** ***n* = 139**	**HCA** **stage II** ***n* = 209**	**HCA** **stage III** ***n* = 116**	**Control vs. HCA stage I**		**Control vs. HCA stage II**		**Control vs. HCA stage III**	
					**AOR (95% CI)^a^**	***P*-value**	**AOR (95%CI)^a^**	***P*-value**	**AOR (95% CI)^a^**	***P*-value**
5-min Apgar score <7	3 (0.7)	0 (0)	0 (0)	1 (0.9)	NA	NA	NA	NA	3.51 (0.12–98.78)	0.462
SGA	4 (0.9)	2 (1.4)	1 (0.5)	3 (2.6)	0.40 (0.01–27.07)	0.671	NA	NA	0.63(0.01–301.94)	0.882
Birth trauma	27 (5.8)	7 (5.0)	15 (7.2)	10 (8.6)	0.97 (0.40–2.37)	0.952	1.20 (0.61–2.37)	0.593	1.52 (0.67–3.43)	0.318
Neonatal jaundice	125 (26.9)	21 (15.1)	61 (29.2)	34 (29.3)	0.47 (0.28–0.80)	0.005	1.15 (0.80–1.67)	0.454	1.13 (0.71–1.81)	0.603
RDS	20 (4.3)	7 (5.0)	5 (2.4)	9 (7.8)	1.18 (0.45–3.08)	0.733	0.46 (0.16–1.33)	0.150	1.85 (0.79–4.34)	0.158
Neonatal pneumonia	9 (1.9)	1 (0.7)	1 (0.5)	2 (1.7)	0.42 (0.04–5.06)	0.494	0.03 (0.01–1.74)	0.091	0.76 (0.13–4.43)	0.758
Neonatal respiratory support	10 (2.2)	5 (3.6)	6 (2.9)	9 (7.8)	1.51 (0.43–5.29)	0.519	1.41 (0.45–4.40)	0.554	4.08 (1.44–11.57)	0.008
NEC	3 (0.7)	1 (0.7)	1 (0.5)	4 (3.5)	0.44 (0.01–16.49)	0.659	NA	NA	NA	NA
Blood transfusion	1 (0.2)	0 (0)	2 (1.0)	1 (0.9)	NA	NA	NA	NA	NA	NA
Neonatal sepsis	7 (1.5)	2 (1.4)	11 (5.3)	8 (6.9)	1.51 (0.22–10.60)	0.676	3.43 (1.21–9.69)	0.020	4.57 (1.34–15.61)	0.015
Admittance to NICU	286 (61.6)	76 (54.7)	146(69.9)	96 (82.8)	0.73 (0.48–1.11)	0.135	1.55 (1.05–2.27)	0.027	3.35 (1.92–5.84)	<0.001
Hospitalization time ≥7 days	99 (21.3)	16 (11.5)	53 (25.4)	42 (36.2)	0.47 (0.26–0.85)	0.012	1.30 (0.87–1.93)	0.200	2.05 (1.29–3.27)	0.002

*HCA, histological chorioamnionitis; AOR, adjusted odds ratio; CI, confidence interval; SGA, small for gestational age; RDS, respiratory distress syndrome; NEC, necrotising enterocolitis; NICU, neonatal intensive care unit; NA, not applicable*.

a *Adjusted for all baseline clinical factors mentioned in PSM*.

## Discussion

The incidence of elevated intrapartum temperature was 4.1% (1,349/33,040) in our study, and the total presence of HCA was 44.5% (601/1,349) among them. In this study, we not only evaluated the differences in the pregnancy outcomes of women with elevated intrapartum temperature complicated by HCA and those with intrapartum temperature ≥37.5°C alone, but also the impacts of different HCA stages.

After PSM and adjusting for various baseline clinical characteristics, we found that women with elevated intrapartum temperature complicated by HCA were more likely to end up with cesarean section, and seemed to have a longer hospitalization time. At the same time, this group of women seemed to have a higher proportion of puerperal morbidity. But further study failed to find an association between HCA stage I and adverse maternal outcomes. We also found an elevated proportion of neonatal sepsis and NICU admission in those with intrapartum temperature ≥37.5°C complicated by HCA. Further analysis suggested that HCA stage II was associated with neonatal sepsis and NICU admission. And for newborns in the HCA stage III group, in addition to the above two outcomes, prolonged hospitalization was observed and the need for respiratory support also increased.

The results of our study were generally in agreement with previous studies of similar design (7, 20). In general, HCA seemed to be related to certain adverse maternal and neonatal outcomes, and advanced HCA stage correlated to worse prognosis. However, there were two abnormal results in the HCA stage I subgroup. According to our study, the combination of HCA stage I might be associated with a decreased proportion of neonatal jaundice and prolonged hospitalization. Previously, only two studies evaluated the association between HCA and neonatal jaundice or phototherapy (21, 22), but neither found a change in the rate of jaundice. Considering that infection is one of the main causes of neonatal jaundice, we further analyzed the relationship between neonatal laboratory results and jaundice, and found that CRP level was positively correlated with the disease (data not shown). Therefore, the reduced proportion of neonatal jaundice might be the result of a relatively lower level of inflammation in the newborns of women with HCA stage I in our study population. In addition, there are big differences in clinical treatment in patients with different severity of jaundice. Physiological jaundice usually does not require special treatment, while pathological jaundice might require pharmacotherapy, phototherapy and even transfusion therapy. In our study, we only analyzed the difference in the incidence of neonatal jaundice, while no statistical analysis on its severity was performed. Therefore, it is possible that after analyzing those who need phototherapy, there might be no significant difference in this outcome between the control groups and the HCA stage I group. As for hospitalization time, although with statistical significance, the difference in the length of hospitalization between the two groups was only half a day (5.17 vs. 4.56 days), therefore, the clinical significance of this result might be relatively limited.

Both the overall analysis and the subgroup analysis indicated that HCA was associated with neonatal sepsis, especially in those with HCA of advanced stage (II-III), which is consistent with the results of many previous studies carried out in preterm infants (23). Augmented neonatal WBC count was also found in the HCA group, suggesting an increase in neonatal inflammation. Similar results have been reported by Han et al. (7), in which the existence of HCA further aggravated the risk of early-onset sepsis in newborns of women with suspected intrauterine infection. It is worth noting that in the subgroup analysis of NICU-admittance (as shown in Figure 5), the preventive use of antibiotics during labor in the HCA group had a tendency to reduce the NICU admission rate [AOR 1.14 (0.75–1.73) vs. 1.69 (1.11–2.57), *p* for interaction = 0.096]. But at the same time, we cannot ignore the fact that there might be differences in the NICU admission standards between different countries and even between different hospitals. Moreover, since it is a retrospective observational study, we are unable to conclude that the use of antibiotics during labor could improve pregnancy outcomes, and a prospective study is necessary to confirm this result.

At the same time, choosing the right type of antibiotic is very important for the prevention of maternal postpartum infection and neonatal infection. As the drug susceptibility test is relatively time-consuming, clinicians usually use antibiotics empirically, and adjust the type of antibiotics according to its effect and the results of the drug susceptibility test later. Our study found that women with HCA had a higher positive rate of mycoplasma in vaginal secretion culture, suggesting that this microorganism might be associated with HCA. This is consistent with previous studies on pathogenic bacteria of chorioamnionitis (11, 24). However, the antibiotics mostly used for the preventive treatment of pregnant women at present are second-generation cephalosporins, which do not have good coverage for mycoplasma (11, 25). In recent years, azithromycin, a macrolide antibiotic, has been used increasingly in the perinatal period. Studies on primate models have confirmed that intravenous azithromycin is effective in eliminating intra-amniotic infection caused by mycoplasma, and can improve pregnancy outcomes such as preterm birth (26, 27). Thus, for women with high suspicions of HCA, the prophylactic use of azithromycin might be effective. But since there were only a small number of women who had a positive result of mycoplasma (65/464 in the HCA group and 34/464 in the control group), and since vaginal secretion culture is not the golden standard for the detection of pathogenic bacteria of HCA (amniotic fluid culture instead), although with statistical significance, we can only regard it as a reference in clinical work. Whether mycoplasma is the pathogenic bacteria of HCA still needs to be confirmed by subsequent studies.

Our study has several strengths. First, to our knowledge, this is one of the few studies that compared the maternal and neonatal outcomes between elevated intrapartum temperature alone and those complicated by HCA. Second, this is a single-center cohort study, so all women and newborns received unified diagnostic criteria and clinical management strategies, and almost all those with hyperthermia underwent placental pathology examination, which ensured the integrity of the retrospective cohort. Meanwhile, we included all full-term single pregnancy women with intrapartum temperature ≥37.5°C after entering active labor during the study period, and only a very small number of women were excluded from our study, which mainly because of incomplete data. Therefore, our study population was almost unselected, which might increase the universality of the results to a certain extent. Additionally, previous study of Haddad et al. (28) found that active labor could increase the expression of genes of inflammation related molecule in fetal membranes, and increased incidence of HCA was observed in those who experienced labor process (12). Thus, in order to eliminate the effect of uterine contraction on the presence of HCA, we only analyzed women who entered the active stage of labor, and those who terminated labor process by cesarean section before cervical dilatation ≥4 cm were excluded from the study, which makes the result more reliable. At the same time, due to the relative lack of research on the effect of HCA on the outcome of full-term pregnancy, our research can provide a reference for clinical work.

There are still some limitations in this study. First, although we included as many confounding factors that might affect pregnancy outcomes as possible, other unmeasured confounding factors might exist and cause bias in the propensity-score matched population. Second, we did not carry out neonate follow-up after hospital discharge, so the long-term neonatal outcomes cannot be traced.

In conclusion, the presence of HCA can further worsen the maternal and neonatal outcomes of full-term women with elevated intrapartum temperature. But women with HCA of stage I shared similar pregnancy outcomes with the control group.

## Data Availability Statement

The original contributions generated in the study are included in the article/Supplementary Material, further inquiries can be directed to the corresponding authors.

## Ethics Statement

The studies involving human participants were reviewed and approved by The Ethic Committee of International Peace Maternity and Child Health Hospital. Written informed consent for participation was not required for this study in accordance with the national legislation and the institutional requirements.

## Author Contributions

YG collected the data, performed the statistical analysis and drafted the manuscript. CZ assisted with the statistical analyses. YC conceptualized and designed the study and was responsible for data collection. HH conceptualized and designed the study, revised the manuscript and gave the final approval of the version to be submitted. All authors contributed to the article and approved the submitted version.

## Conflict of Interest

The authors declare that the research was conducted in the absence of any commercial or financial relationships that could be construed as a potential conflict of interest.
