# Supplementary material for: Adverse Maternal and Neonatal Outcomes in Women With Elevated Intrapartum Temperature Complicated by Histological Chorioamnionitis at Term: A Propensity-Score Matched Study
Source: Front Pediatr. 2021 Apr 22;9:654596. doi: 10.3389/fped.2021.654596 (PMC8344350; doi:10.3389/fped.2021.654596)
Supplement: Supplementary file 1 [file Data_Sheet_1.docx]

**Supplementary Table 1.** Univariable regression analysis of maternal outcomes in the propensity-score matched cohort^a^.

|  | **Control group** | **HCA group** | **OR (95%CI)** | **P value** |
| --- | --- | --- | --- | --- |
|  | **n=464** | **n=464** |  |  |
| **Mode of delivery** |  |  |  |  |
| Eutocia | 306 (66.0) | 276 (59.5) | Reference |  |
| Forceps/Vacuum | 100 (21.6) | 108 (23.3) | 1.20 (0.87-1.64) | 0.265 |
| Cesarean section | 58 (12.5) | 80 (17.2) | 1.53 (1.05-2.23) | 0.026 |
| **Placental adhesion** | 17 (3.7) | 20 (4.3) | 1.18 (0.61-2.29) | 0.615 |
| **Cervix or vagina laceration** | 11 (2.4) | 7 (1.5) | 0.63 (0.24-1.64) | 0.345 |
| **Postpartum hemorrhage** | 13 (2.8) | 19 (4.1) | 1.48 (0.72-3.04) | 0.283 |
| **Puerperal morbidity** | 14 (3.0) | 37 (8.0) | 2.79 (1.49-5.23) | 0.001 |
| **Late postpartum hemorrhage** | 3 (0.7) | 1 (0.2) | 0.33 (0.03-3.20) | 0.340 |
| **Admittance to ICU** | 9 (1.9) | 9 (1.9) | 1.00 (0.39-2.54) | 1.000 |
| **Hospitalization time≥7days** | 85 (18.3) | 122 (26.3) | 1.59 (1.16-2.18) | 0.004 |

HCA: histological chorioamnionitis; OR: odds ratio; CI: confidence interval; ICU: intensive care unit.

^a^ Data is presented as frequency (percentage).

**Supplementary Table 2.** Univariable regression analysis of neonatal outcomes in the propensity-score matched cohort^a^.

|  | **Control group** | **HCA group** | **OR (95%CI)** | **P value** |
| --- | --- | --- | --- | --- |
|  | **n=464** | **n=464** |  |  |
| **5-min Apgar score <7** | 3 (0.7) | 1 (0.2) | 0.33 (0.03-3.20) | 0.340 |
| **SGA** | 4 (0.9) | 6 (1.3) | 1.51 (0.42-5.37) | 0.528 |
| **Birth trauma** | 27 (5.8) | 32 (6.9) | 1.20 (0.71-2.04) | 0.502 |
| **Neonatal jaundice** | 125 (26.9) | 116 (25.0) | 0.90 (0.67-1.21) | 0.501 |
| **RDS** | 20 (4.3) | 21 (4.5) | 1.05 (0.56-1.97) | 0.873 |
| **Neonatal pneumonia** | 9 (1.9) | 4 (0.9) | 0.44 (0.13-1.44) | 0.174 |
| **Neonatal respiratory support** | 10 (2.2) | 20 (4.3) | 2.05 (0.95-4.42) | 0.069 |
| **NEC** | 3 (0.7) | 6 (1.3) | 2.01 (0.50-8.10) | 0.325 |
| **Neonatal sepsis** | 7 (1.5) | 21 (4.5) | 3.10 (1.30-7.35) | 0.011 |
| **Blood transfusion** | 1 (0.2) | 3 (0.7) | 3.01 (0.31-29.07) | 0.340 |
| **Admittance to NICU** | 286 (61.6) | 318 (68.5) | 1.36 (1.03-1.78) | 0.028 |
| **Hospitalization time≥7days** | 99 (21.3) | 111 (23.9) | 1.16 (0.85-1.58) | 0.347 |

HCA: histological chorioamnionitis; OR: odds ratio; CI: confidence interval; SGA: small for gestational age; RDS: respiratory distress syndrome; NEC: necrotising enterocolitis; NICU: neonatal intensive care unit.

^a^ Data is presented as frequency (percentage).

**Supplementary Table 3.** Maternal outcomes in the subgroup of intrapartum temperature ≥38℃^a^.

|  | **Control group** | **HCA group** | **OR (95%CI)** | **aOR (95% CI)** | **P value** |
| --- | --- | --- | --- | --- | --- |
|  | **n=140** | **n=143** |  |  |  |
| **Mode of delivery** |  |  |  |  |  |
| Eutocia | 78 (55.7) | 79 (55.2) | Reference | Reference |  |
| Forceps/Vacuum | 36 (25.7) | 31 (21.7) | 0.85 (0.48-1.51) | 0.86 (0.46-1.62) | 0.642 |
| Cesarean section | 26 (18.6) | 33 (23.1) | 1.25 (0.69-2.29) | 1.41 (0.74-2.65) | 0.295 |
| **Placental adhesion** | 4 (2.9) | 7 (4.9) | 1.75 (0.50-6.12) | 2.64 (0.55-12.62) | 0.225 |
| **Cervix or vagina laceration** | 6 (4.3) | 16 (11.2) | 2.81 (1.07-7.42) | 5.37 (1.58-18.27) | 0.007 |
| **Postpartum hemorrhage** | 5 (3.6) | 6 (4.2) | 1.18 (0.35-3.97) | 1.49 (0.33-6.69) | 0.603 |
| **Puerperal morbidity** | 3 (2.1) | 4 (2.8) | 1.31 (0.29-5.98) | 2.85 (0.36-22.31) | 0.318 |
| **Late postpartum hemorrhage** | 0 (0) | 0 (0) | NA | NA | NA |
| **Admittance to ICU** | 1 (0.7) | 2 (1.4) | 1.97 (0.18-21.99) | NA | NA |
| **Hospitalization time≥7days** | 32 (22.9) | 44 (30.8) | 1.50 (0.88-2.55) | 1.73 (0.98-3.07) | 0.061 |

HCA: histological chorioamnionitis; OR: odds ratio; aOR: adjusted odds ratio; CI: confidence interval; ICU: intensive care unit; NA: not applicable.

^a^ Data is presented as frequency (percentage).

**Supplementary Table 4.** Neonatal outcomes in the subgroup of intrapartum temperature ≥38℃^a^.

|  | **Control group** | **HCA group** | **OR (95%CI)** | **aOR (95% CI)** | **P value** |
| --- | --- | --- | --- | --- | --- |
|  | **n=140** | **n=143** |  |  |  |
| **5-min Apgar score <7** | 2 (1.4) | 0 (0) | NA | NA | NA |
| **SGA** | 1 (0.7) | 1 (0.7) | 0.98 (0.06-15.81) | NA | NA |
| **Birth trauma** | 10 (7.1) | 13 (9.1) | 1.30 (0.55-3.07) | 1.31 (0.53-3.25) | 0.561 |
| **Neonatal jaundice** | 46 (32.9) | 35 (24.5) | 0.66 (0.39-1.11) | 0.66 (0.38-1.14) | 0.136 |
| **RDS** | 10 (7.1) | 6 (4.2) | 0.57 (0.20-1.61) | 0.47 (0.15-1.52) | 0.206 |
| **Neonatal pneumonia** | 5 (3.6) | 2 (1.4) | 0.38 (0.07-2.01) | 0.24 (0.03-2.19) | 0.206 |
| **Neonatal respiratory support** | 5 (3.6) | 8 (5.6) | 1.60 (0.51-5.02) | 2.24 (0.53-9.40) | 0.272 |
| **NEC** | 2 (1.4) | 1 (0.7) | 0.49 (0.04-5.42) | NA | NA |
| **Neonatal sepsis** | 1 (0.7) | 9 (6.3) | 9.34 (1.17-74.70) | NA | NA |
| **Blood transfusion** | 1 (0.7) | 0 (0) | NA | NA | NA |
| **Admittance to NICU** | 13 (9.3) | 15 (10.5) | 1.15 (0.52-2.50) | 1.17 (0.50-2.73) | 0.720 |
| **Hospitalization time≥7days** | 117 (83.6) | 119 (83.2) | 0.98 (0.52-1.82) | 1.00 (0.50-2.00) | 0.996 |

HCA: histological chorioamnionitis; OR: odds ratio; aOR: adjusted odds ratio; CI: confidence interval; SGA: small for gestational age; RDS: respiratory distress syndrome; NEC: necrotising enterocolitis; NICU: neonatal intensive care unit; NA: not applicable.

^a^ Data is presented as frequency (percentage).

**Supplementary Table 5.** Maternal outcomes in the control group and the CCA group

|  | **Control group** | **CCA group** | **OR (95%CI)** | **aOR (95% CI)** | **P value** | |
| --- | --- | --- | --- | --- | --- | --- |
|  | **n=614** | **n=180** |  |  |  |  |
| **Mode of delivery** |  |  |  |  | |  |
| Eutocia | 412 (67.1) | 101 (56.1) | Reference | Reference | |  |
| Forceps/Vacuum | 126 (20.5) | 35 (19.4) | 1.00 (0.54-1.83) | 0.67 (0.35-1.27) | | 0.216 |
| Cesarean section | 76 (12.4) | 44 (24.4) | 2.30 (1.34-3.95) | 1.94 (1.08-3.49) | | 0.027 |
| **Placental adhesion** | 17 (2.8) | 5 (2.8) | 1.00 (0.37-2.76) | 1.13 (0.34-3.79) | | 0.843 |
| **Cervix or vagina laceration** | 19 (3.1) | 12 (6.7) | 2.24 (1.06-4.70) | 2.08 (0.84-5.13) | | 0.114 |
| **Postpartum hemorrhage** | 27 (4.4) | 13 (7.2) | 1.69 (0.85-3.35) | 1.31 (0.57-3.04) | | 0.529 |
| **Puerperal morbidity** | 10 (1.6) | 3 (1.7) | 1.02 (0.28-3.76) | 1.83 (0.31-10.89) | | 0.507 |
| **Late postpartum hemorrhage** | 4 (0.7) | 1 (0.6) | 0.85 (0.10-7.67) | 0.15 (0.01-8.26) | | 0.355 |
| **Admittance to ICU** | 15 (2.4) | 7 (3.9) | 1.62 (0.65-4.03) | 3.18 (1.02-9.94) | | 0.046 |
| **Hospitalization time≥7days** | 113 (18.4) | 44 (24.4) | 1.43 (0.97-2.13) | 1.32 (0.83-2.09) | | 0.246 |

HCA: histological chorioamnionitis; OR: odds ratio; aOR: adjusted odds ratio; CI: confidence interval; ICU: intensive care unit.

^a^ Data is presented as frequency (percentage).

**Supplementary Table 6.** Neonatal outcomes in the control group and the CCA group

|  | **Control group** | **CCA group** | **OR (95%CI)** | **aOR (95% CI)** | **P value** |
| --- | --- | --- | --- | --- | --- |
|  | **n=614** | **n=180** |  |  |  |
| **5-min Apgar score <7** | 2 (0.3) | 0 (0) | NA | NA | NA |
| **SGA** | 5 (0.8) | 0 (0) | NA | NA | NA |
| **Birth trauma** | 34 (5.5) | 13 (7.2) | 1.33 (0.69-2.57) | 1.23 (0.58-2.58) | 0.591 |
| **Neonatal jaundice** | 154 (25.1) | 34 (18.9) | 0.70 (0.46-1.05) | 0.75 (0.47-1.19) | 0.223 |
| **RDS** | 26 (4.2) | 6 (3.3) | 0.78 (0.32-1.93) | 0.91 (0.31-2.71) | 0.869 |
| **Neonatal pneumonia** | 10 (1.6) | 2 (1.1) | 0.68 (0.15-3.13) | 1.65 (0.21-12.93) | 0.633 |
| **Neonatal respiratory support** | 16 (2.6) | 5 (2.8) | 1.07 (0.39-2.96) | 1.66 (0.49-5.62) | 0.420 |
| **NEC** | 4 (0.7) | 1 (0.6) | 0.85 (0.10-7.67) | 0.46 (0.03-6.34) | 0.561 |
| **Neonatal sepsis** | 7 (1.1) | 6 (3.3) | 2.99 (0.99-9.01) | 3.95 (1.01-15.55) | 0.049 |
| **Blood transfusion** | 1 (0.2) | 0 (0) | NA | NA | NA |
| **Admittance to NICU** | 360 (58.6) | 106 (59.6) | 1.04 (0.74-1.46) | 1.16 (0.76-1.77) | 0.499 |
| **Hospitalization time≥7days** | 120 (19.5) | 30 (16.7) | 0.82 (0.53-1.28) | 1.13 (0.69-1.87) | 0.629 |

HCA: histological chorioamnionitis; OR: odds ratio; aOR: adjusted odds ratio; CI: confidence interval; SGA: small for gestational age; RDS: respiratory distress syndrome; NEC: necrotising enterocolitis; NICU: neonatal intensive care unit; NA: not applicable.

^a^ Data is presented as frequency (percentage).
